# Sequencing-Based Genotyping of Pakistani *Burkholderia mallei* Strains: A Useful Way for Investigating Glanders Outbreaks

**DOI:** 10.3390/pathogens11060614

**Published:** 2022-05-24

**Authors:** Hanka Brangsch, Muhammad Saqib, Awais ur Rehman Sial, Falk Melzer, Jörg Linde, Mandy Carolina Elschner

**Affiliations:** 1 Institute for Bacterial Infections and Zoonoses, Friedrich-Loeffler-Institute, Naumburger Str. 96a, 07743 Jena, Thuringia, Germany; falk.melzer@fli.de (F.M.); joerg.linde@fli.de (J.L.); mandy.elschner@fli.de (M.C.E.); 2 Veterinary Preventive Medicine and Public Health Laboratory, Department of Clinical Medicine and Surgery, University of Agriculture, Faisalabad 38000, Pakistan; drsaqibm@uaf.edu.pk; 3 Department of Clinical Studies, Faculty of Veterinary and Animal Science, Pir Mehr Ali Shah, Arid Agriculture University Rawalpindi, Rawalpindi 46000, Pakistan; awais_1717@yahoo.com

**Keywords:** glanders, *Burkholderia mallei*, SNP typing, cgMLST scheme, genotyping, Pakistan, WGS

## Abstract

*Burkholderia* (*B.*) *mallei* is a host-adapted equine pathogen that causes glanders, a re-emerging zoonotic disease, which is endemic in Pakistan and other developing countries and seriously impacts the global equine movement. Due to globalization, the geographical restriction of diseases vanishes and the lack of awareness of and experience with eradicated diseases in industrialized countries also promotes the re-introduction of infections in these regions. Owing to the high equine population, the Pakistani province Punjab is a potential hotspot where several glanders outbreaks have been seen over last two decades. For determining the genomic diversity of *B. mallei* in this and other equine-populated prefectures, the genomes of 19 *B. mallei* strains isolated between 1999 and 2020 in different locations were sequenced and their genotypes were determined. Particularly, for genetically highly homogenous pathogens like *B. mallei* genotyping techniques require a high discriminatory power for enabling differentiation on the strain level. Thus, core-genome single nucleotide polymorphism (cgSNP) analysis was applied for distinguishing the highly similar strains. Furthermore, a whole-genome sequence-based core genome multi locus sequence typing (cgMLST) scheme, specific to *B. mallei*, was developed and additionally applied to the data. It was found that *B. mallei* genotypes in Pakistan persisted over time and space and genotype clusters preferred connection with a time point rather than the place of isolation, probably due to frequent equine movement, which promotes the spread of glanders. The cgMLST approach proved to work in accord with SNP typing and may help to investigate future glanders outbreaks.

## 1. Introduction

International animal trading poses the risk of global dissemination of pathogens. Even on smaller scales (i.e. animal movement between districts), unrecognised infection carriers can have a fatal impact, causing outbreaks among the native population [1]. Such transmission scenarios have occurred for glanders, a bacterial infection caused by *Burkholderia mallei* that mainly affects equines [2,3]. Horses, in particular, can develop an asymptomatic, chronic form of glanders, which makes them perilous spreaders, while mules and donkeys usually decease rapidly due to acute glanders [4]. Although solipeds are its primary host, *B. mallei* can also infect other mammals, including humans, making it a zoonotic pathogen. Laboratory workers, veterinarians and animal caretakers are at highest risk [5,6]. There is no vaccine available and antibiotic treatment of glanders is laborious and protracted [7,8].

In the past, glanders received wide attention due to the fatal nature of the disease, but now it has been eradicated in many countries due to strict measurements and culling policy [9]. However, sporadic cases still occur, often attributed to animal import from regions where glanders is endemic, e.g. Africa, Greater Middle East, Asia and South America. Rising numbers of *B. mallei* infection led to the classification of glanders as a re-emerging disease [9,10,11,12].

One of the earliest documentations of glanders in Pakistan dates back to 1877 and several outbreaks have been reported since the beginning of the present century [13,14,15]. Hornstra, Pearson, Georgia, Liguori, Dale, Price, O’Neill, Deshazer, Muhammad, Saqib, Naureen and Keim [1] showed that 15 strains isolated between 1999 and 2007 can be classified into three distinct lineages, which persist over decades and geographic distances. However, serological studies on potentially undetected glanders infection in equids in Punjab, a Pakistani province with a large equine population, did not detect seropositive animals, and it was assumed that few local foci must exist from where *B. mallei* is sporadically disseminated to other equine populations by asymptomatic animals [15,16]. Additionally, many owners do not cull glanderous animals after detection of the infection due to the high value of the animals and low indemnity, thereby promoting the persistence of glanders in developing countries [17,18,19].

The differentiation of *B. mallei* strains on a molecular basis and therefore the tracing of infection sources is complicated, as the global population of this pathogen is genetically highly homogenous [20]. Several methods for molecular typing of *B. mallei* are at hand, most of which were originally developed for the closely related *B. pseudomallei* making them hardly applicable for *B. mallei*. In a multi locus sequence typing (MLST) scheme most *B. mallei* belong to a single genotype, as the chosen target genes are highly conserved [21]. Variable numbers of tandem repeat analyses [22] provide a higher resolution than MLST. However, global population analysis based on tandem repeat regions can be impaired by homoplasy [23]. Thus, two methods of choice for comparing bacterial genotypes on a larger scale are single nucleotide polymorphism (SNP) typing and core genome MLST (cgMLST), where changes in a larger proportion of the genomes are considered.

SNP typing has rarely been applied for the differentiation of *B. mallei* yet. Girault, et al. [24] used 15 informative SNPs that revealed three lineages in the global *B. mallei* population, which was later confirmed by whole genome SNP analysis [25]. Furthermore, wgSNPs showed that two distinct *B. mallei* populations caused outbreaks of glanders in Bahrain in 2010 and 2011 [26]. Allel-based methods such as cgMLST use a standardized nomenclature by indexing different allelic states of target genes (“targets”) and are an interesting alternative to SNP-based approaches often with comparable results [27,28]. In fact, cgMLST is an expansion of the classical MLST scheme to a genome-wide approach, which provides a high resolution due to thousands of target genes. In that way, local outbreak strains were differentiated, as well as global population structures examined for several pathogens, e.g., for *Acinetobacter baumanii*, *Bacillus anthracis* and *Listeria monocytogenes* [29,30,31].

In this study we aim at elucidating the diversity of *B. mallei* genotypes circulating in Pakistan based on whole genome sequencing. By developing a *B. mallei*-specific cgMLST scheme, we add an additional method to the epidemiologist’s toolbox for determining connections between outbreak events and infection chain tracing.

## 2. Results

### 2.1. Strain Isolation and Identification

Between 2017 and 2020, eight *B. mallei* strains (Table 1) were recovered from clinically suspected cases of glanders in game (polo) and draught equid communities from different areas of Pakistan (Figure S1). The identity as *B. mallei* was confirmed by PCR targeting *bimA_ma_*.

For investigating whether there is a prevalence of certain genotypes in Pakistan, we also included a panel of *B. mallei* strains (*n* = 11) isolated from different outbreaks of glanders between 1999 and 2007 (Table 1). These strains were isolated from clinical samples of puss, blood and nasal swabs of equines that were used as working animals, e.g., in the police service or polo matches.

### 2.2. Genome Sequencing

The Pakistani strains were sequenced by Illumina technology yielding on average 2,419,160 reads (range: 1,615,876–3,957,054) per sample with an average length of 255 bp and sufficiently high coverage for further analysis (Table 2, Table S1). Genomes that were assembled from these reads met the expected size and GC content. However, due to the short-read sequencing approach, the genomes remained fragmented, comprising 262 to 379 contigs (Table 2).

In contrast, by pursuing a hybrid assembly strategy combining Illumina short-read and nanopore long-read sequencing data, a higher level of contiguity could be reached for the genomes of four strains from our strain collection (Table 3).

### 2.3. SNP Typing of Pakistani Strains

**Figure 1:**
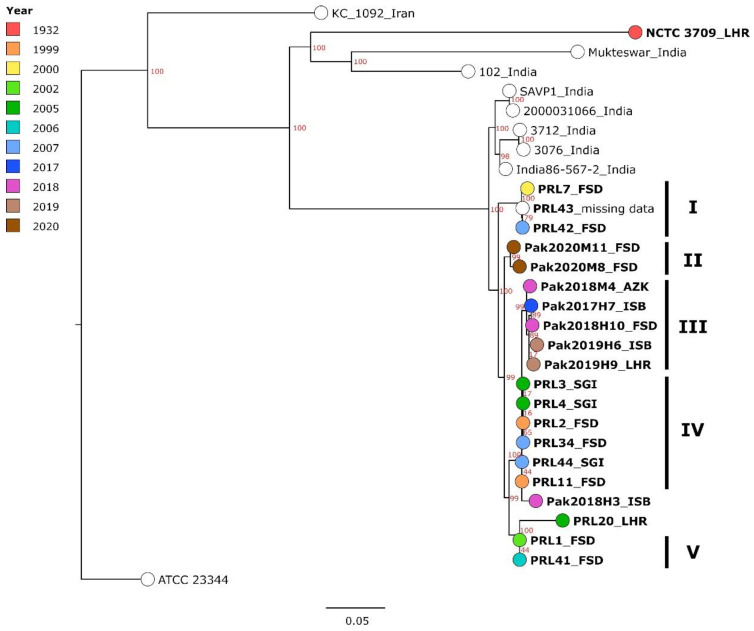
Maximum-likelihood tree, generated based on cgSNPs called by Snippy. Strains from Pakistan are printed in bold with the district of isolation given after the name (LHR—Lahore; FSD—Faisalabad; AZK—Azad Jammu and Kashmir; ISB—Islamabad; SGI—Sargodha) and the year of isolation indicated by color. For non-Pakistani strains, the country of isolation is given. The bar indicates base substitutions per site. Clusters formed by Pakistani strains are denoted by Roman numerals. Numbers in red represent bootstrap support values.

The investigated strains from Pakistan were compared in a cgSNP analysis (Figure 1, Table S2) to seven strains from India, one historic strain from Pakistan and one from Iran, isolated between 1932 and 2015. In this analysis, 1016 core genome SNPs were called. The recent strains from Pakistan formed one large cluster that clearly differentiated them from a cluster formed by Indian strains by at least 35 SNPs. The historic strain NCTC 3709, isolated in 1932 in Lahore, did not fall within the Pakistani cluster, exhibiting 327 to 367 SNP differences compared to the contemporary strains. 

Within the Pakistani cluster, the strains formed smaller separate clusters, which were defined by sampling decade rather than by location of isolation, i.e., the strains from 2017 to 2020 did not mix in clusters with the PRL-named strains (1999–2007). In a cgSNP analyses merely including the strains sequenced in this study, all in all, 660 core genome SNPs were detected and SNP differences of 0–96 SNPs were observed (Table S2). In the polytomy based on these cgSNP data, which was in agreement with Figure 1 (Figure S2), five Pakistani clusters could be made out (Figure 1), although within these, the SNP distance could be as high as 22 SNPs (Pak2020M11 and Pak2020M8 forming cluster II). The most homogenous cluster, cluster IV, was formed by strains from Faisalabad and Sargodha between 1999 and 2007 with 0–3 differing SNPs. Furthermore, PRL1 and PRL41 (cluster V), both isolated in Faisalabad but with a four-year distance, exhibited identical cgSNP profiles. The assembly of strain PRL20, a strain that had already been sequenced and published before [1], was also included in the genotyping analysis. The distance between this strain and the cluster formed by PRL1 and PRL41 constituted 35 SNPs.

It was striking that the more contemporary strains exhibited fewer clusters, with less than 10 varying SNPs, implying a higher genomic heterogeneity of strains from 2017 to 2020. The strains from Faisalabad (2018; Pak2018H10) and Islamabad (2017; Pak2017H7) were the most similar among these strains, as they were separated merely by two SNP, while all other strains exhibited at least 10 SNP differences. They belonged to the most prominent cluster, cluster III, formed by strains from 2017 to 2019 from almost every sampled location. When comparing the two sampling decades, the strains exhibited 10 to 96 SNPs difference.

### 2.4. cgMLST Scheme Development and Validation

Due to the high quality criteria for penetration query genomes as a basis for the cgMLST scheme, several strains commonly used in *B. mallei* genotyping studies were not represented in the set of chosen public database entries. Thus, we additionally sequenced four strains of our strain collection (Table 3) using Illumina short-read in combination with nanopore long-read sequencing for high-quality hybrid assemblies that were added to the set of genomes as the basis for cgMLST development. All in all, 22 genomes were chosen for cgMLST generation (Table S1), none of which were identified as taxonomic or quality outlier. Using the cgMLST Target Definer, 2838 of 5025 genes were identified as suitable targets for the scheme (56.5% of the reference genome), while 1890 genes (37.6%) were classified as accessory. Further 297 genes (5.9%) were discarded as a result of the Multi Copy Filter analysis. Thus, the final scheme comprised 2838 genes.

**Figure 2:**
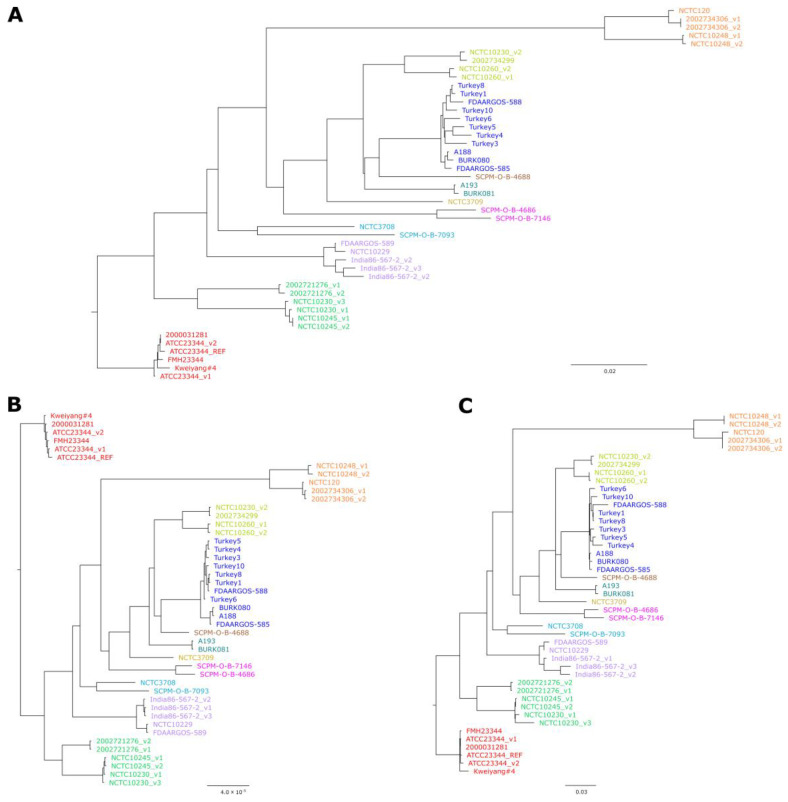
Comparison between trees generated by different approaches: (**A**) Neighbor-Joining tree based on cgMLST allelic profiles using 2838 target genes; (**B**) Maximum-likelihood tree based on cgSNP alignment generated by Snippy using Illumina read data; (**C**) Approximately Maximum-likelihood tree based on cgSNP alignment generated with Parsnp using genome assemblies. For convenience, clusters or singletons of strains that showed in the trees were colored identically. Bars indicate allelic changes (**A**) or base substitutions per site (**B**,**C**). Bootstrap values for (**B**,**C**) can be found in Figure S3.

In order to validate the newly defined scheme, sequences of 47 *B. mallei* strains representing the currently known genomic diversity, including sequences of the same strains from multiple sources, were analyzed by cgMLST and compared to the results of the cgSNP analysis, which is the current gold standard. For this cgSNP analysis, a read- and assembly-based approach were chosen, using the tools Snippy and Parsnp. The neighbor-joining analysis based on cgMLST profiles was very well in accordance with both trees based on cgSNP data (Figure 2, Figure S3).

On average, 97.24% of the cgMLST targets were called from these assemblies, with a mean value of 98.1%, although the number of contigs of these assemblies ranged from 209 to 1382. In three tested assemblies, less than 75% of target genes were identified, thus they were excluded from the analysis (Table S1). These assemblies also displayed the lowest N50 values of all tested data, <6600 bp, and they were highly fragmented, comprising >1600 contigs.

Despite varying percentages of good targets, there were also duplicate strains from different sequencing projects clustered together that displayed 0–27 allelic differences in cgMLST. It was observed that an N50 value below 15,000 bp markedly reduced the number of identifiable targets to less than 95%.

For one strain, NCTC10230, the allelic profiles between one out of three datasets differed immensely from the other two, namely in 306 and 329 targets. Accordingly, the strains clustered differently in the tree, which was also the case in the trees based on SNP analysis. Thus, it must be concluded that the strain name was incorrectly assigned to this sequence data.

In the cgSNP analysis 2318 SNPs were called by Snippy and 2676 by Parsnp, depicting the variability between both approaches. Accordingly, the number of differing SNPs between strains varied with the methods. The distances calculated by cgMLST lay within the ranges of the SNP calling tools (Table S3).

### 2.5. Allele-Based Typing of Pakistani Strains

The Pakistani strains were subjected to cgMLST analysis using the newly developed cgMLST scheme. On average, 98.87% of the targets were called (97.2–99.3%; Table S1). In this analysis, no identical allelic profiles were detected and the strains differed in 1–87 targets (Table S4). In agreement with the cgSNP analysis, the more recent Pakistani strains displayed higher heterogeneity (13–73 targets) than the older strains (1–55 targets). 

**Figure 3:**
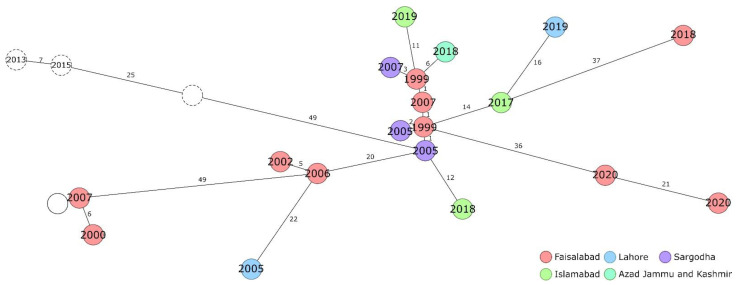
Minimum-spanning tree based on cgMLST allelic profile differences. The Pakistani strains are coloured according to the place of isolation with the year of isolation given. Circles with dotted lines represent strains isolated in India. Numbers on the branches indicate the number of allelic differences. The empty, solid line circle represents strain PRL43, for which no metadata was available.

The most homogenous cluster found in the cgSNP analysis, cluster IV, also showed in the cgMLST results (Figure 3). These strains exhibited 1–4 allelic differences and formed a cluster in the center of the tree, around which branches with the more contemporary strains emerged. Thereby, in contrast to the cgSNP analysis, the cluster that was formed by five strains from 2017 to 2019, SNP cluster III, was dispersed and the congruence of Pak2017H7, Pak2018M4 and Pak2019H6 to strains from police horses from Faisalabad (1999) became more apparent. Furthermore, existing differences between some of these strains were more pronounced. Especially the strain Pak2018H10 (Faisalabad, 2018) clearly was differentiated from the others by 37 to 73 alleles. Likewise, the two strains from 2019 from Lahore and Islamabad, Pak2019H6 and Pak2019H9, did not group together in the cgMLST analysis, although the difference of 20 alleles was well in accordance with the 20 SNPs difference in cgSNP analysis. 

Furthermore, strains PRL1 and PRL41, both from Faisalabad but isolated four years apart, could be differentiated by cgMLST profiles (five alleles of a difference), which was not possible by cgSNP (zero SNP differences). However, the PRL and more recent strains still did not form clusters defined by isolation location or year. The three exemplary Indian strains that were included in the cgMLST analysis did not mix with Pakistani strains.

Additionally, in silico multiple-locus variable number tandem repeat analysis (MLVA) was conducted for the Pakistani strains (Table S5). For some of the strains investigated here, MLVA profiles have been published before [1]. When the in silico profiles were compared to those, the allelic numbers for several loci diverged from these published profiles. Thus, in silico MLVA was dismissed for genotyping as the assembly of the target regions, which exhibit a high number of repeats, pose a particular challenge to assemblers and the MLVA data based on PCR was considered more reliable.

## 3. Discussion

Although glanders has been endemic in Pakistan since at least the 19th century, little is known about the distribution of different *B. mallei* genotypes in this region. The present study is the first extensively employing whole genome sequencing for molecular genotyping of *B. mallei* outbreak strains from Pakistan. Often, typing studies suffer from the availability of only a few strains that can be investigated, which might pose a problem for revealing the true genomic variability within this specie and determining the method that is best suited for differentiation of more distantly related strains as well as highly congruent outbreak isolates. Thus, we investigated 19 strains that cover a sampling period of over two decades and originate from different locations in Pakistan.

For a reliable differentiation of species and strains, it is necessary to identify unique molecular signatures with a high discriminatory power. Several methods are at hand. However, the *Burkholderia*-specific MLST scheme [21] fails to differentiate the highly clonal *B. mallei* strains while an MLVA scheme [22] provides higher resolution, but the investigated repeat regions are prone to homoplasy. Recently, a cgMLST scheme for *B. pseudomallei*, the assumed progenitor of *B. mallei*, was proposed [32]. As this scheme could also not sufficiently differentiate *B. mallei* strains (unpublished data), a *B. mallei*-specific cgMLST scheme was developed in the present study. The percentage of targets identified as suitable core genome genes included in this scheme was well in range with other studies, although this value is highly specie-dependent. The number of identified target genes, 2838 targets, was lower than the published number of 3456 *B. mallei* core genome genes [20]. However, for the definition of the core genome in the present study, more strains (22 strains) were used than before (seven strains, [20]) and it is known that with an increasing number of genomes included in the analysis, the size of the detected core genome decreases [33].

In a well-defined cgMLST scheme the retrieval rate of these targets in outbreak strains should constitute at least on average 95% to 97.5% [29,31]. This was the case for the *B. mallei* scheme when challenged with sequences from worldwide strains covering the complete diversity of the species, as well as the Pakistani outbreak strains. Likewise, the new scheme was compatible with cgSNP analysis as both revealed the same epidemiological patterns. However, Pakistani strains that were identical in the SNP analysis showed differences in cgMLST allelic profiles. Thus, we believe the cgMLST scheme might help outbreak investigations, in which highly congruent strains have to be differentiated. For other species, thresholds of allelic differences have been determined that define a single outbreak event, e.g., five and twenty alleles in case of *Bacillus anthracis* and *Enterococcus faecium*, respectively [27,30]. As in the present study, the true epidemiologic connection between isolates remained elusive, and further studies are required to determine this value for *B. mallei*. Such a threshold is also not known yet for *B. pseudomallei* cgMLST analyses, as even two alleles separate outbreak strains of a single transmission event from unrelated isolates and epidemiological connections were merely assumed for isolates differing by one allele [32]. A similarly strict differentiation could be expected for *B. mallei*.

When the newly developed cgMLST scheme was employed for the analysis of a diverse set of global *B. mallei* strains, the resulting polytomy matched the known patterns and was in accordance with the cgSNP typing results. The analysis of duplicate strains from different sequencing projects deposited in public databases gave allelic patterns that for some strains differed in several targets. However, differences can be attributed to the age of the isolates resulting in different replication cycles in the laboratories, as well as varying sequence qualities. Observed differences in allelic numbers of one and the same strain could be attributed to ambiguities in the assemblies. In particular, the N50 value of the assemblies proved crucial for the success of cgMLST analysis. In fragmented assemblies, genomic elements might be truncated or missing. In order to overcome this lack of high-quality assemblies for the development of the cgMLST scheme, we added genome sequences of four strains to the set of query genomes. The assemblies for those strains were generated by a hybrid assembly approach combining Illumina and nanopore sequencing data, which improved genome completeness and accuracy.

Although glanders is endemic in Pakistan and neighboring countries for at least 150 years [13,14,15,18], there are rarely whole genome sequence data present in the public databases. This complicates epidemiological investigations that rely on the determination of genome similarity for tracking strain origins. Serological studies proved the prevalence of *B. mallei* in the Punjab province [34], to which Faisalabad, Sargodha and Lahore belong, although glanders seems to be restricted to local endemic points [15].

The whole genome genotyping approaches employed in this study showed that the Pakistani strains form a group within global *B. mallei* phylogeny that can be distinguished from closely located Indian strains. Apparently, there is no extensive mixing between *B. mallei* populations from both countries.

The *B. mallei* PRL strains have been investigated before by MLVA [1], on the basis of which three clusters could be defined (clades A–C). The largest of these clusters, clade A, contains amongst others the strains PRL3, PRL4, PRL11 and PRL44, which were also clustered in this study by both cgSNP (cluster IV) and cgMLST analyses. According to Hornstra et al. [1], the hosts to PRL3, PRL4 and PRL44 originate from the same farm. Two horses, hosts to the former two strains, got infected with *B. mallei* in Lahore, which might be the source of infection of a mule, host to PRL44, 1.5 years later. However, the MLVA profiles of the strains differ [1]. In the presented study these strains were highly similar in cgSNP as well as the cgMLST profile, suggesting an epidemiological connection. As repeat regions are less stable markers than SNPs, the time distance between the isolation might account for the differing MLVA profiles. It has to be remarked, that strains from 1999 (PRL2, PRL11) and 2007 (PRL34) also fell into cluster IV in the present study, as they exhibited a similar level of identity to those strains (0–3 SNPs and 1–4 cgMLST alleles differences), although no connection between the cases is known.

When comparing the cgSNP and cgMLST data, strains that differed merely by a couple or by no SNPs, although they were isolated from different places and/or years apart, showed larger differences in cgMLST profiles, as would be expected and this makes sense from an epidemiological point of view, as comparably high numbers of genome alterations occur in *B. mallei* during passage through a host [35]. The same source persisting over eight years for these strains is unlikely, although horses are known to carry chronic glanders infections for several years [10]. PRL 1 and PRL 41, that were identical in cgSNP analysis, differed in MLVA profile [1] as well as in cgMLST alleles. Furthermore, PRL2 and PRL3, which could neither be differentiated by MLVA [1] nor by SNP typing, showed a slightly different allelic cgMLST profile, which would be expected as they were isolated in 1999 and 2005 in Faisalabad and Sargodha, respectively. Thus, cgMLST can add information and might help differentiating strains compared to SNP and MLVA analysis. We can confirm the former notion [1] that numerous *B. mallei* lineages circulate in Pakistan. However, the connection between genomic links and epidemiological links remains difficult given the complex epidemiological situation where horses often move between different cities and regions.

The analysis is complicated by a gap of 11 years between the sampling periods and by the fact that various undetected glanders outbreak events can be expected in the region [14]. In prefectures with high equine population, glanders is still prevalent and outbreaks in breeding establishments, as observed in 2006 to 2007 in Sargodha [15] might support the spread of *B. mallei* throughout the country. However, the currently circulating strains are distinct from NCTC 3709, isolated at the beginning of the 20th century in Lahore. It is known that glanders was re-introduced in India in the 1960s during the Indo-China war by imported horses and also employed in warfare [10,18], which might be the reason for genotype differences between the historic and the currently circulating *B. mallei* strains in Pakistan.

Thorough genotype characterization of *B. mallei* strains would not only help to elucidate the origin of Pakistani strains, but also the detection and surveillance of glanders worldwide. Laroucau et al. [36] reported that in two horses that were serologically tested and found to be positive for glanders, the routine PCR with tissue targeting the *fliP*-IS407A gene did not give a positive result, probably due to genetic variation in the infecting strains. Solely by applying more extensive methods, like SNP and MLST typing, the serological results could be confirmed. For such cases, in silico analysis based on whole genome sequences might help improving diagnostics, which is also important for countries where glanders is eradicated for decades, as veterinarians do not recognize the symptoms of the disease easily [11]. The application of cgMLST that gives a lab-independent, uniform sequence type assignment could further support the tracing of the origins of infections.

Here, we could show that the read-dependent cgSNP approach works well together with cgMLST analysis that is based on assemblies, and we believe that future studies could benefit from employing both methods, especially when the analysis must be based on assemblies, when no raw read data is available.

## 4. Materials and Methods

### 4.1. Sampling and Identification

*B. mallei* strains were isolated from blood and puss samples of clinically suspected glanders equids (horses and mules), which were brought to the Veterinary Medical Teaching Hospital (VMTH) of the University of Agriculture (UAF), Pakistan, between 1999 and 2020. Blood cultures were carried out in Oxoid Signal Blood Culture System (Oxoid, Basingstoke, UK). For this purpose, approximately 10 mL peripheral venous blood collected from the jugular vein was inoculated and incubated on a shaker at 37 °C for 56 h. Samples where the indicator devices showed positive culture signals were sub-cultured on 5% sheep blood agar plates and presumptive colonies were biochemically tested and confirmed by conventional PCR targeting the *B. mallei bimAma* gene as described elsewhere [37]. The amplicons were cleaned and sequenced by Lab-Genetix (Lahore, Pakistan) for further confirmation. Puss samples were cultured on blood agar plates and incubated at 37 °C for 56 h. Whitish grey to grey, non-hemolytic or marginally hemolytic colonies were also confirmed by PCR as described above.

### 4.2. Cultivation and DNA Isolation

For DNA isolation, the Pakistani *B. mallei* strains were grown in brain-heart infusion broth containing 4% glycerol at 37 °C for 2d. DNA was extracted using enzymatic digestion and phenol-chloroform extraction, according to standard protocols [38].

For the development of a cgMLST scheme covering a high diversity of *B. mallei*, four *B. mallei* strains from the Friedrich-Loeffler Institutes’ strain collection (Mukteswar, NCTC120, 34, BfR242) were selected, for which no high-quality genome assemblies were deposited in the public databases, yet. The strains were grown on nutrient agar (Merck, Darmstadt, Germany) supplemented with 3% glycerine and 7.5% blood for 48 h at 37 °C. DNA was isolated using the NucleoBond HMW DNA kit (Macherey-Nagel, Düren, Germany) and subsequently used for Illumina and nanopore sequencing library preparation.

### 4.3. Library Preparation, Sequencing and Assembly

Short-read sequencing libraries were prepared from the isolated DNA with the Nextera XT library preparation kit (Illumina Inc., San Diego, CA, USA) and subsequently sequenced using v3 chemistry on a MiSeq system (Illumina) in paired-end mode. 

Four strains (Mukteswar, NCTC120, 34, BfR242) were additionally sequenced by nanopore long-read technology (ONT). For this purpose, libraries were prepared with the Ligation Sequencing Kit SQK-LSK 109 (Oxford Nanopore Technologies Ltd., Oxford, UK) together with the Barcoding Kit EXP-NBD 104 (Oxford Nanopore Technologies Ltd., Oxford, UK) and sequenced on an R9.4.1 flow cell with a MinION Mk1B sequencing device (Oxford Nanopore Technologies Ltd., Oxford, UK) for 24 h. Sequencing raw data and hybrid assemblies were deposited at the European Nucleotide Archive under project number PRJEB52165.

### 4.4. Raw Data Processing

Basecalling and demultiplexing of the ONT data were conducted with Guppy basecaller v5.0.7 (Oxford Nanopore Technologies Ltd: Oxford, UK, 2021) applying the “super-accuracy” model. The read quality was checked by NanoPlot v1.32.1 [39]. Finally, by using Unicycler v0.4.8 [40], hybrid assemblies from long- and corresponding short-read data were generated. 

The Illumina reads were assembled using Shovill v1.0.4 (assembler: SPAdes; https://github.com/tseemann/shovill, accessed on 11 April 2022). Short reads and assemblies were analyzed using the pipeline WGSBAC v2.2 (https://gitlab.com/FLI_Bioinfo/WGSBAC/, accessed on 11 April 2022) including a check for species identity and contaminations by kraken2 [41] as well as read and assembly quality assessment by FASTQC v0.11.7 (https://www.bioinformatics.babraham.ac.uk/projects/fastqc/, accessed on 11 April 2022) and Quast v5.0.2 [42], respectively. Coding genomic regions were annotated by Prokka v1.14.5 [43]. 

### 4.5. Genotyping

Furthermore, core genome SNP typing with Snippy v.4.6.0 (https://github.com/tseemann/snippy, accessed on 11 April 2022), as well as 23-loci MLVA [22] using MISTReSS (https://github.com/Papos92/MISTReSS, accessed on 11 April 2022) were conducted utilizing WGSBAC. *B. mallei* ATCC 23344 (GCF_000011705.1) was used as reference strain for SNP typing. Trees were visualized with FigTree v1.4.3 (http://tree.bio.ed.ac.uk/software/figtree/, accessed on 11 April 2022) and figures were made publication-ready using Inkscape v1.1 (https://inkscape.org, accessed on 11 April 2022). For further genotyping by cgMLST using a scheme generated in the framework of this study, Ridom SeqSphere+ v7.7 [44] was used and minimum spanning tree based on the allelic profiles was generated with the parameter “pairwise ignore missing values” for distance calculation.

Additionally, the public Sequence Read Archive (SRA) and NCBI GenBank were browsed on 20 January 2022 for Illumina read data and assemblies of *B. mallei* strains isolated in Pakistan and neighboring countries. This foreign data was processed using WGSBAC as described above. Accession numbers and metadata of foreign strains, as well as their function in this study, can be found in Table S1.

### 4.6. cgMLST Scheme Generation and Validation

Public databases were searched for genome assemblies of *B. mallei* on 11 October 2021. Metadata and function of these sequences are listed in Table S1. The quality of the assemblies was assessed using the WGSBAC pipeline (see above). For the generation of the cgMLST scheme 18 publicly available genomes were chosen (Table S1) as they met the following criteria: sequences covered at least 89% of the genome fraction of the reference strain ATCC 23344 (GCF_000011705.1), showed less than 2 N’s per 100 kb, no contaminations and the number of contigs did not exceed 20. For doublet strains, the assembly with the highest quality was chosen. Four hybrid assemblies generated in the course of the present study (see above) were included in the set of strains used for cgMLST scheme development.

The cgMLST scheme was generated by a genome-wide gene-by-gene comparison using the MLST^+^ target definer incorporated in Ridom SeqSphere+ v7.7 with default parameters, as previously described [31], including several quality filters to ensure scheme stability. The genome of *B. mallei* ATCC 23344 (GCF_000011705.1) served as the seed genome.

For validation of the scheme, publicly available *B. mallei* raw reads from the NCBI SRA database were downloaded (accessed on 20 October 2021; Table S1) and assembled with Shovill v1.0.4. The assemblies were analyzed with the new cgMLST scheme. A gene was considered a good target if it aligned with 100% to the reference sequence and showed at least 90% identity to this reference. A distance tree was calculated based on the allelic profiles by neighbor-joining algorithm implemented in SeqSphere+ v7.7 with pairwise ignoring missing values. By default, only samples with at least 90% of detected targets were included in the analysis. Furthermore, a core genome SNP analysis based on the raw reads and assemblies was conducted by Snippy v.4.6.0 (https://github.com/tseemann/snippy, accessed on 11 April 2022) in conjunction with RAxML v8.2.12 [45] and Parsnp v1.2 within the Harvest suite [46], respectively. The resulting trees were compared using Dendroscope v3.5.9 [47]. Bootstrapping was performed whenever possible using RAxML with 200 iterations.

## Figures and Tables

**Table 1:** Metadata of *B. mallei* strains isolated in Pakistan from 1999 until 2020.

Strain	Year	Source	Host	Population	Region	Purpose
Pak2018H3	2018	Blood	Horse	Private farm	Islamabad	Polo
Pak2018M4	2018	Pus	Mule	Sample received for confirmation	Azad Jammu and Kashmir	Draught
Pak2019H6	2019	Pus	Horse	Private owner having total 28 imported polo ponies	Islamabad	Polo
Pak2017H7	2017	Blood	Horse	Private	Islamabad	Polo
Pak2020M8	2020	Blood	Mule	For hauling	Faisalabad	Draught
Pak2019H9	2019	Blood	Horse	Owner has 40 polo ponies	Lahore	Polo
Pak2018H10	2018	Blood	Horse	Cart horse	Faisalabad	Draught
Pak2020M11	2020	Blood	Mule	For hauling	Faisalabad	Draught
PRL1	2002	Pus	Donkey	For hauling	Faisalabad	Draught
PRL2	1999	Nasal swab	Horse	Police service	Faisalabad	Mounted Police Horse
PRL3	2005	Pus	Horse	Private	Sargodha	Farm
PRL4	2005	Pus	Horse	Private	Sargodha	Farm
PRL7	2000	Pus	Horse	For hauling	Faisalabad	Draught
PRL11	1999	Pus	Horse	Police service	Faisalabad	Mounted Police Horse
PRL34	2007	Nasal swab	Donkey	Work in brick factory	Faisalabad	Draught
PRL41	2006	Pus	Mule	For hauling	Faisalabad	Draught
PRL42	2007	Pus	Mule	For hauling	Faisalabad	Draught
PRL43	NA	NA	NA	NA	NA	NA
PRL44	2007	Nasal swab	Mule	Private	Sargodha	Farm

**Table 2:** Assembly quality data of the investigated Pakistani strains, which were sequenced using the Illumina short-read technique.

Strain	Coverage	Bases	Contigs	GC (%)	L50	N50	GF * (%)	CDS
Pak2018H3	100	5,526,644	295	68.68	44	43,202	92.86	4614
Pak2018M4	118	5,526,233	269	68.68	41	46,366	92.95	4615
Pak2019H6	79	5,526,261	262	68.69	40	46,511	92.98	4631
Pak2017H7	86	5,528,440	272	68.69	42	46,377	92.97	4623
Pak2020M8	98	5,593,509	284	68.22	41	46,937	92.64	4639
Pak2019H9	68	5,305,987	266	68.60	40	44,187	89.23	4442
Pak2018H10	75	5,536,192	307	68.65	45	43,210	92.91	4630
Pak2020M11	127	5,530,694	379	68.59	61	30,209	92.59	4667
PRL1	121	5,523,415	294	68.66	43	43,660	92.95	4610
PRL2	112	5,512,370	302	68.67	43	42,887	92.54	4595
PRL3	116	5,599,466	279	68.69	40	46,978	92.50	4680
PRL4	120	5,517,077	287	68.68	41	43,747	92.54	4605
PRL7	114	5,282,618	281	68.58	41	43,782	88.62	4432
PRL11	124	5,509,016	290	68.68	41	45,145	92.56	4600
PRL34	90	5,559,549	287	68.73	42	45,246	92.51	4623
PRL41	82	5,589,007	272	68.71	39	46,976	92.87	4666
PRL42	78	5,575,591	270	68.70	38	48,213	93.81	4652
PRL43	82	5,579,744	271	68.69	41	46,808	93.82	4658
PRL44	171	5,527,185	294	68.69	42	43,055	92.95	4613

* Genome fraction covering reference genome ATCC 23344.

**Table 3:** Assembly quality data of hybrid assemblies using Illumina short-read in conjunction with ONT long-read techniques.

Strain	Bases	Contigs	L50	N50	GF * (%)	CDS
34	5,647,473	1	1	5,647,473	94.62	4812
Mukteswar	5,760,320	11	1	3,539,038	96.27	4909
BfR 242	5,375,480	18	1	3,503,053	90.00	4632
NCTC 120	5,401,604	19	1	4,027,971	89.47	4668

* Genome fraction covering reference genome ATCC 23344.

## Data Availability

The data presented in this study are openly available in ENA BioProject PRJEB52165 and in the Supplementary Materials.

## Supplementary Materials

The following supporting information can be downloaded at: https://www.mdpi.com/article/10.3390/pathogens11060614/s1, Figure S1: Map of Pakistan and sampling locations (LHR—Lahore; FSD—Faisalabad; AZK—Azad Jammu and Kashmir; ISB—Islamabad; SGI—Sargodha). The map is property of Wikimedia Commons and licensed under the Creative Commons Attribution-Share Alike 3.0 Unported license (https://creativecommons.org/licenses/by-sa/3.0/deed.en, accessed on 12 April 2022) and was modified; Figure S2: Maximum likelihood tree based on cgSNP alignment of exclusively Pakistani strains using Snippy; Figure S3: Tanglegrams of cgMLST- and cgSNP-based trees. Red numbers indicate bootstrap support values; Table S1: Foreign data accession data and assembly quality of Pakistani strains and foreign data; Table S2: SNP distances between Pakistani strains; Table S3: cgMLST allele distances of strains used for scheme validation; Table S4: cgMLST allele distances of Pakistani strains; Table S5: In silico MLVA profiles of Pakistani strains.
