# Supplementary figures and images for: Sequencing-Based Genotyping of Pakistani Burkholderia mallei Strains: A Useful Way for Investigating Glanders Outbreaks
Source: Pathogens. 2022 May 24;11(6):614. doi: 10.3390/pathogens11060614 (PMC9227068; doi:10.3390/pathogens11060614)

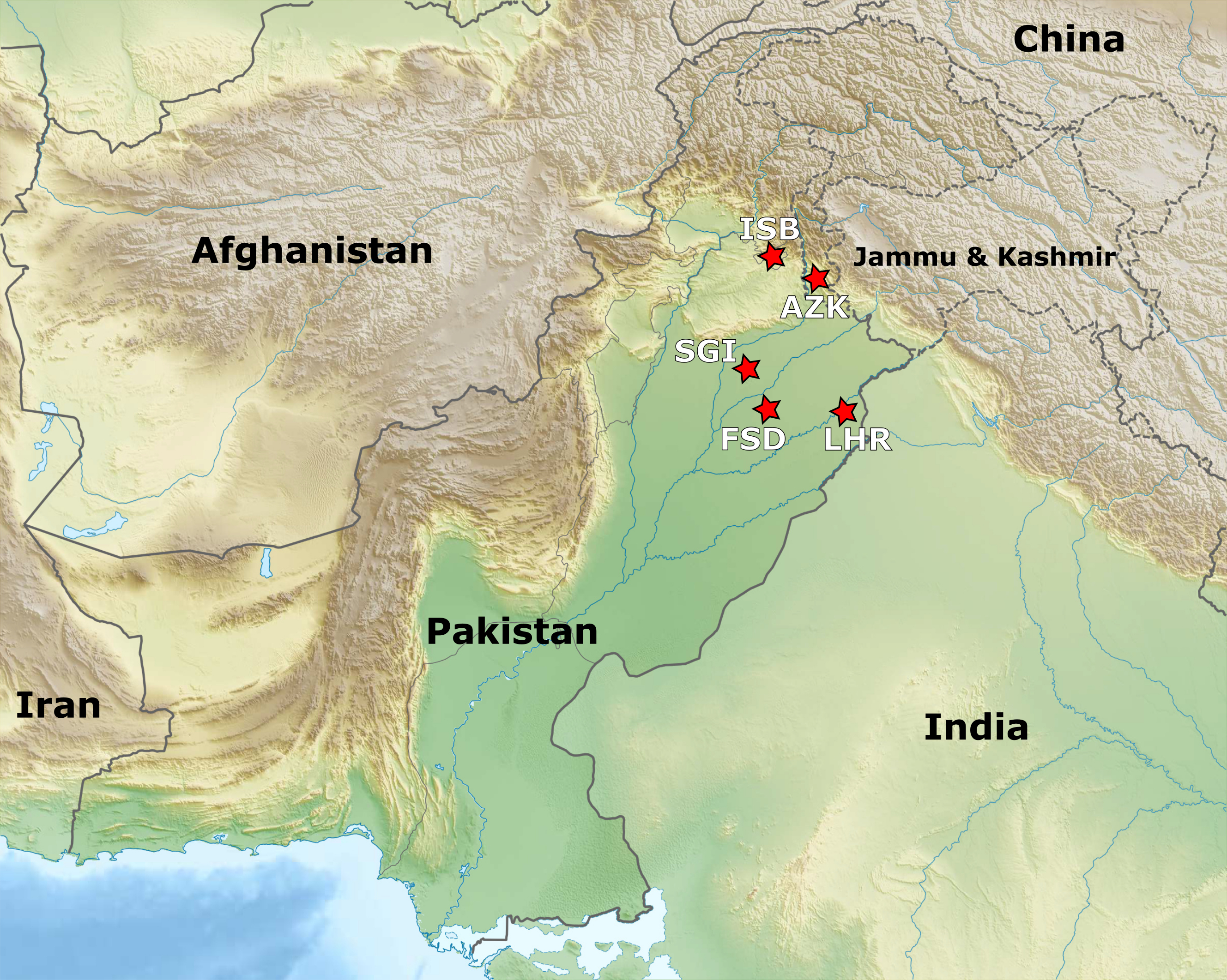

Supplement: Supplementary file 1 [file pathogens-11-00614-s001.zip › Supplement Figure S1 - Map of Pakistan and sampling locations .jpg]

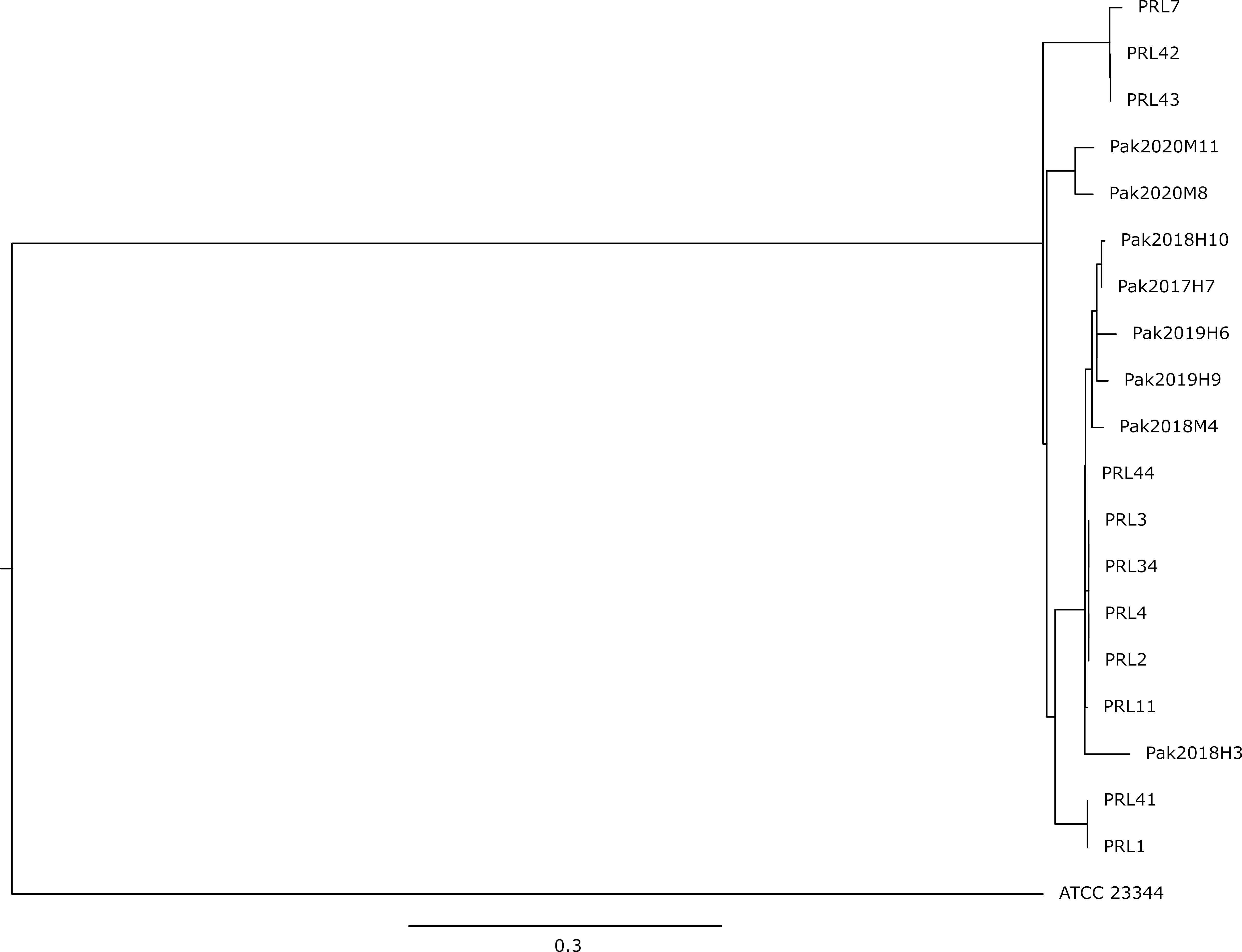

Supplement: Supplementary file 1 [file pathogens-11-00614-s001.zip › Supplement Figure S2 - Maximum likelihood tree based on cgSNP alignment of exclusively Pakistani strains using Snippy.jpg]
